# Supplementary material for: Tead1a Initiates Transcriptional Priming Through the TEAD1a/YAP‐Notch1‐Spi1/Cebpα Axis to Promote Neutrophil Fate
Source: Adv Sci (Weinh). 2025 Aug 28;12(41):e05441. doi: 10.1002/advs.202505441 (PMC12591127; doi:10.1002/advs.202505441)
Supplement: Supplementary file 4 — Supplementary Dataset [file ADVS-12-e05441-s004.zip › 3-6.zip!/3-6/02-6.cluster/cluster16/cluster16_dotplot.pdf]

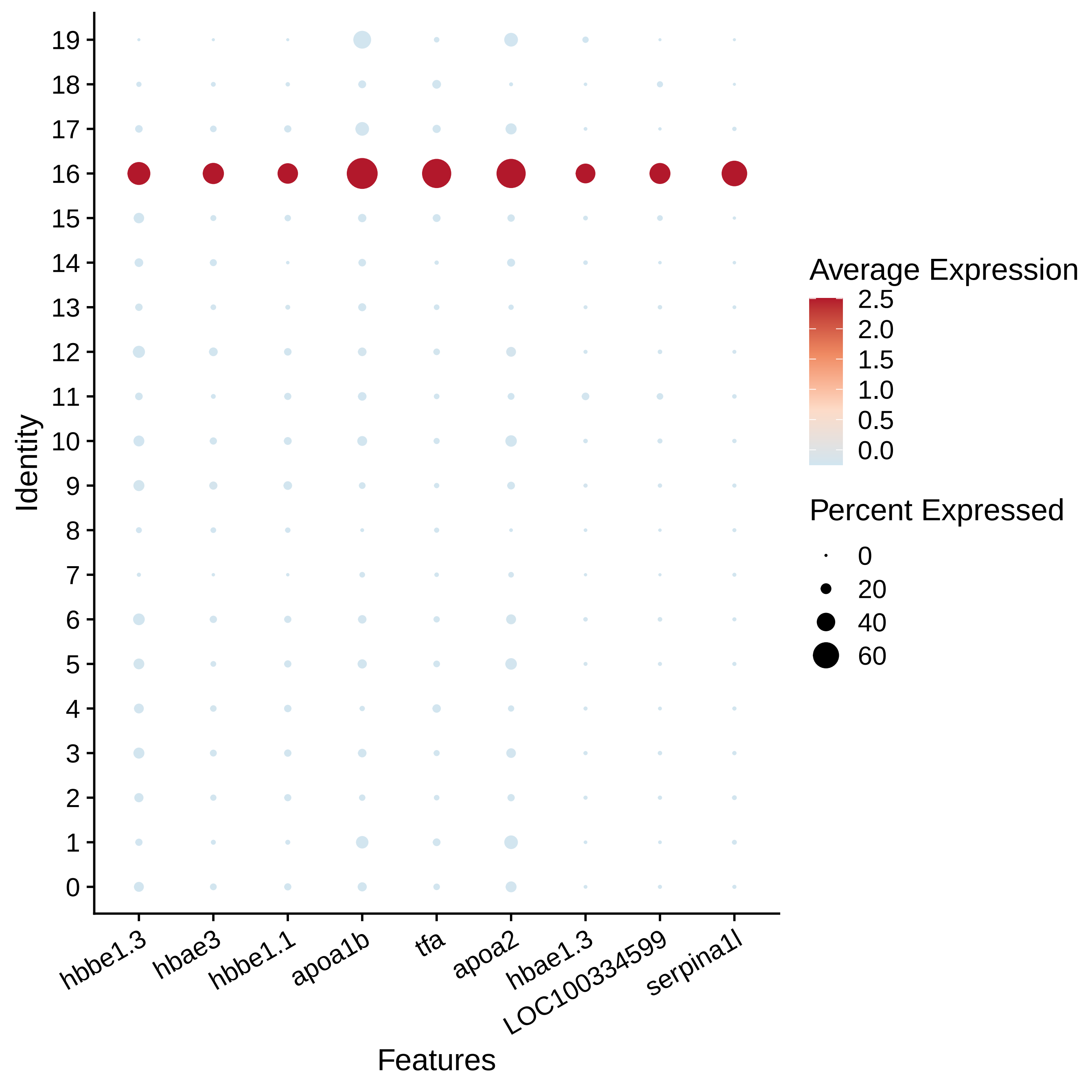

Average Expression
2.5
2.0
1.5
1.0
0.5
0.0
Percent Expressed
0
20
40
60
Identity
19
18
17
16
15
14
13
12
11
10
9
8
7
6
5
4
3
2
1
0
Features
hbbe1.3
hbae3
hbbe1.1
apoa1b
tfa
apoa2
hbae1.3
LOC100334599
serpina1l
